# Speckle-Tracking Echocardiography - Ready for Use in Acute Coronary Syndrome?

**DOI:** 10.5935/abc.20180061

**Published:** 2018-04

**Authors:** Brivaldo Markman Filho

**Affiliations:** Hospital das Clínicas da Universidade Federal de Pernambuco, Recife, PE - Brazil

**Keywords:** Acute Coronary Syndrome, Speckle Tracking, Strain, Echocardiography, Doppler, Risk Factors/prevalence, Echocardiography

Population aging and the increase of risk factors such as arterial hypertension and diabetes, mainly associated with obesity, have greatly contributed to increased hospitalizations of patients with acute coronary syndrome (ACS).^1,2^ Patients with ACS may have different prognosis and, for this reason, risk stratification of ACS, including unstable angina (UA), is mandatory.^3,4^ In this context, anatomical definition of culprit artery using coronary cineangiography and percutaneous intervention has been the first choice for patients at moderate-to-high risk.^5^ Doppler echocardiography has a key role at the emergency room to assess left ventricle function and to rule out other conditions that may influence the diagnosis.^6^

Recently, the use of two-dimensional speckle-tracking echocardiography (2D-STE) for measurement of myocardial strain has gained importance for its applicability in the clinical practice.^7^ Its high sensitivity to measure systolic function and identify left ventricular subclinical dysfunction, as compared with left ventricular ejection fraction, extends its applicability and makes it a test of additional value in many areas of cardiology.^8^

Despite promising data, 2D-STE has not been sufficiently standardized as a routine method for the diagnosis of myocardial ischemia. Characteristics inherent to the technique affect its applicability in both acute and chronic phase of the ischemic event, as previous ventricular deformities may affect the interpretation of results.^9^

The study by dos Santos et al.^10^ provides us with a pioneer study on the real applicability of left ventricular longitudinal strain in UA. The authors describe the frequency at which 2D-STE is indicated in cardiac emergencies and evaluate the values of the test in patients with severe coronary artery lesions. We highlighted some interesting findings of this study.

The authors assessed 78 patients with clinically suspected UA, and found that 2D-STE was indicated in less than 20% of the patients. History of infarction or percutaneous intervention were the main limitations for the use of the technique in more than half of the sample. These findings highlight the limitation of the method in the assessment of coronary disease in emergency situations.

The authors then compared eligible and non-eligible patients for 2D-STE and did not find a pattern of association between the applicability of the test and the clinical variables measured, except for the frequency of diabetes, which was significantly higher in the non-eligible group. Although the power of the test is limited in this study design, this finding could raise the hypothesis that the presence of diabetes, usually associated with a more severe prognosis, could represent a limitation for application of the method.

Another interesting finding in this series was the accuracy of 2D-STE. The presence of severe coronary lesions was confirmed by coronary cineangiography in most of the fifteen patients considered eligible for 2D-STE. Besides, the authors observed that global strain was significantly reduced in patients with severe lesions in any epicardial coronary artery and that the longitudinal strain was significantly reduced in the basal segments of left ventricular inferior and lateral walls of the right and circumflex coronary arteries. It is of note that there was no association between myocardial strain and severe lesion in anterior descending artery, probably due to the small sample size, as reported by the authors. These findings corroborate the current evidence showing that reduced (global and segmental) strain is correlated with the severity of myocardial ischemia in terms of the number of coronary vessels affected.^11^

In their conclusion, the authors suggested that 2D-STE can help in the decision-making process of patients in emergency care for investigation of coronary disease. However, based on current knowledge, further studies are still needed to recommend the 2D-STE in the routine clinical practice. Until that happens, caution is needed, and indication of this feasible but still not formally recommended method should be carefully considered in cardiology emergency centers of both private and public services.
